# Parents' mental state language and child gender: A scoping review of developmental evidence

**DOI:** 10.1111/bjdp.12502

**Published:** 2024-06-06

**Authors:** Callyn Farrell, Ellen Sanderson, Aisling Mulvihill, Michael Thai, Virginia Slaughter

**Affiliations:** ^1^ School of Psychology The University of Queensland Brisbane Queensland Australia

**Keywords:** child development, gender differences, language, mental state language, parents, scoping review

## Abstract

Mental state language (MSL) is an important mechanism through which children learn about their social world and place within it. Previous research has suggested that parents may use MSL differently towards children based on their child's gender. However, findings are inconsistent. This scoping review explores the consistency of reported differences in parents' MSL use as a function of children's gender while exploring the methodological variables that may provide insights into these differences. Based on a review of the 27 studies included, 12 found a significant relationship between child gender and parents' MSL, while the remaining did not. The included studies used a range of methodological approaches to elicit MSL. This scoping review allows researchers and practitioners to reflect upon assumptions regarding the associations between child gender and parents' MSL. Further, we call for the use of diverse and informed approaches when studying these associations from a developmental perspective in the future.

**Statement of Contribution**

**What is already known on this subject?**

- Previous research has suggested that parents may use MSL differently towards boys and girls.
- However, the findings are inconsistent and have not been reviewed to date.

**What the present study adds?**

- This scoping review allows researchers and practitioners to reflect upon assumptions regarding the association between child gender and parents' MSL.
- Suggestions are made regarding using diverse and informed approaches when studying associations between child gender and parental MSL from a developmental perspective in the future.

## INTRODUCTION

Parental interactions with their children are influenced by their child's gender. This is evident in the parental selection of material objects (e.g., toys, clothing) and birth names for their children (Coltrane & Adams, 2008; Lytton & Romney, 1991). Research also shows that parents use language differently towards children as a function of the child's gender. For example, Farrell et al. (2023) found that parents preferred to use more elaborative linguistic responses when responding to boys or girls, compared to gender‐neutral children. Additionally, Crowley et al. (2001) demonstrated that, in a naturalistic setting, mothers and fathers of both boys and girls offered increasingly scientific linguistic explanations to boys compared to girls. Further, several investigations have demonstrated that mothers show a preference for increased language use with girls compared to boys and that the content of the linguistic exchanges between mothers and girls is generally more supportive and elaborated (Leaper et al., 1998; Reese & Fivush, 1993; Reese & Newcombe, 2007).

Sociocultural and ecological accounts suggest that these gendered parenting approaches may reflect gender stereotypes and beliefs evident in the wider social world. Such practices may contribute to the gender socialization of children (Bronfenbrenner & Morris, 1998; Leaper, 2002; Leaper & Friedman, 2007). It is well‐established that the frequency, content and quality of language use with children has implications for their cognitive and social development (Anderson et al., 2021; Lucca et al., 2019; Song et al., 2014; Tompkins et al., 2018). Therefore, it is important to consider if and how parents may vary features of their language use towards children as a function of their child's gender.

The present scoping review focuses on parents' use of mental state language (MSL) towards children. MSL is a language that refers to mental states such as cognitions, desires and emotions (Tompkins et al., 2018). While MSL has been referred to in several different ways throughout the literature – for example, internal state language, internal state talk and mental state talk – we use the term MSL to represent linguistic mentions of cognitions, desires and emotions in the present review as per the operationalization presented by Tompkins et al. (2018). A social constructivist account posits that linguistic exchanges about mental states are crucial for children's development of social understanding (e.g., Symons et al., 2006; Tompkins, 2015; Vygotsky, 1978). That is, children gain an understanding of their own and others' mental states through participation in discourse about the mind (Nelson et al., 2003; Symons et al., 2006; Taumoepeau & Ruffman, 2006). When parents use MSL in conversation with their children, they provide an opportunity for children to connect mental states and human behaviour, thus informing an understanding of their own and others' minds (Slaughter & Peterson, 2012). Language plays an important role in social contexts as it allows children to represent social interactions with others before participating in these interactions later. Further, MSL labels and draws attention to the mental states of ourselves and others, which for children, may increase their interest and understanding of mental states within social contexts. This subsequently may allow children to better understand how specific behaviours or cues represent internal mental states (Christensen & Michael, 2016; Scott, 2017).

A robust literature has demonstrated that parental MSL directed towards children is positively associated with children's theory of mind (ToM). ToM is our human ability to infer mental states (e.g., thoughts, intentions, beliefs, desires) and to use these inferences to predict and explain behaviour (see Ruffman, 2023; Slaughter, 2015; Tompkins et al., 2018 for reviews). Associations exist between parents' MSL and their children's ToM, including longitudinally (e.g., Adrián et al., 2007; Ensor & Hughes, 2008; Meins et al., 2003; Ruffman et al., 2002), simultaneously (e.g., Howard et al., 2008; LaBounty et al., 2008; Peterson & Slaughter, 2003), via self‐report (Peterson & Slaughter, 2003), within laboratories (Ruffman et al., 2002) and in naturalistic contexts (Howard et al., 2008). Meta‐analyses have also explored the associations between parental MSL and children's ToM, as well as the association between parental MSL and children's emotion understanding (e.g., Tompkins et al., 2018). Given the role of ToM in forming successful social interactions and relationships (Hughes & Dunn, 1998; Imuta et al., 2016; Slaughter et al., 2015), parental MSL has implications for children's social success. From a social constructivist perspective, the MSL directed towards children accounts for some individual variability in children's ToM development (Hughes & Devine, 2015). Furthermore, evidence suggests that linguistic references to emotions account for some variability specifically in children's emotion understanding (Garner et al., 1997).

Considering the pervasive impact of children's gender on parental behaviour and language (Coltrane & Adams, 2008; Crowley et al., 2001; Leaper et al., 1998; Lytton & Romney, 1991; Reese & Fivush, 1993; Reese & Newcombe, 2007), an important but unanswered question is whether or not parents' MSL varies as a function of their child's gender. How parents use MSL with their children may be a gendered process, whereby parents discuss mental states differently based on the gender of their child (e.g., Farrell et al., 2023). If a child's gender influences parents' MSL use, this may have implications for that child's subsequent development of social cognition and social skills. Further, the association between parents' MSL use and a child's gender may represent an additional mechanism by which children's gender is socialized and gender stereotypes are perpetuated. For instance, if the use of MSL abundant in cognitive words within STEM contexts is predominantly reserved for boys, it could potentially impact and cultivate their interest in STEM, thus reinforcing the stereotype that boys excel in discussions and activities within such domains.

The body of research presented in this review explores the association between child gender and parents' MSL use, however, we expect that the operationalization of gender and/or sex will rarely be explicitly defined or distinguished in the included studies, despite the differences between gender and sex having been acknowledged for some time (see Fausto‐Sterling et al., 2012, for a review). Gender refers to an individual's feelings, thoughts, attitudes and behaviours, and is considered a social identity that is socially constructed. In contrast, sex refers to the biological sex assigned to a person, typically at birth (American Psychological Association, 2020). In modern Western contexts, gender and sex have often been thought of as binary, and a person's gender is often assumed to be congruent with their sex. However, empirical explorations into gender and sex have illuminated the complex relationship between these factors. Emerging work highlights the potential for incongruence between gender and sex, as well as the diversity and fluidity of sexual and gender identities (e.g., Morgenroth & Ryan, 2021).

The present scoping review aims to synthesize and discuss the findings of previous research investigating the associations between child gender and parents' MSL use. Additionally, we aim to review how previous studies have operationalized gender and MSL (and whether they account for gender diversity), explore what motivated the investigation of associations between child gender and parental MSL, catalogue the methodological variations of these studies and identify any consistencies among the findings.

## METHOD

### Information sources

PsycINFO, Scopus, ERIC and Google Scholar were searched. Reference lists of included sources were screened for relevant studies. Unpublished theses were not included in this review. Where possible, a restriction on publication year was set at 1982 due to Bretherton and Beeghly's formative publication on MSL (Bretherton & Beeghly, 1982). Detailed search terms, filters and limits are available in Appendix S1. Additionally, a list of included study references is available in Appendix S2. In short, search terms centred around MSL and those producing it.

### Inclusion and exclusion criteria

To best serve the aim of this review, only studies that had a specific aim or focus on investigating or understanding child gender associations with MSL were included (i.e., more than a preliminary statistical check of gender differences). We included empirical studies published in English and conducted with children aged 12 or younger. The child cut‐off age of 12 years was applied due to the general acceptance that children older than 12 years have entered adolescence and the focus of this review was parents’ use of MSL towards children. Studies were required to have at least one measure of spoken parental MSL, as opposed to written or self‐reported. While self‐reported measures of MSL are available, these may be subject to self‐report bias and may not accurately capture the spoken MSL that parents actually use during conversational interactions with their children. For example, one commonly used self‐report MSL measure, the Maternal Mental State Input Inventory (Peterson & Slaughter, 2003), includes a series of vignettes and pre‐constructed responses that parents rank in order of preference. However, the gender of the protagonists represented within the vignettes is not necessarily matched to the parent participant's child. Additionally, the MSL from a parent and another individual (e.g., child or sibling) cannot have been combined into a single measure of overall MSL. As parental MSL may vary when children present with a developmental disability (Hutchins et al., 2017; Slaughter et al., 2007), the review only included studies with typically developing children. We limited the included studies to those where MSL was spoken in English due to cross‐cultural differences in parents' use of MSL (e.g., Chang et al., 2017; Doan et al., 2019; Kong et al., 2023; Taumoepeau et al., 2019). When datasets were used for several individual studies, the earliest study was the only one included, so a single dataset and the findings of that dataset were represented in the review only once. Included studies were required to report the gender of parent and child participants, and the context used to elicit MSL.

### Context of the current review

Established review practices published by Arksey and O'Malley (2005) were followed to conduct the current scoping review, develop the research question, identify relevant studies, select included studies, collect the data and collate, summarize and report the results of the review (see Arksey & O'Malley, 2005). A scoping review was deemed the most appropriate review methodology for several reasons. Of the 27 studies that met the criteria for inclusion in this review, only 10 included the required statistical information to perform effect size analyses regarding gender differences in parents' MSL use. Of these 10 studies, nine reported significant differences between the MSL used by parents towards girls compared to boys (Martin & Green, 2005). In contrast, 17 studies did not provide the required statistical information, and these 17 included mostly non‐significant findings in regard to child gender. Hence, the data available was heavily biased towards significant differences in the MSL offered to girls and boys by parents. Regarding the 17 studies that provided no data, requests were sent to the corresponding authors, of which two replied with the required data. Additionally, there was considerable heterogeneity across these studies in terms of whose MSL was measured (e.g., mothers, fathers), how MSL was elicited (e.g., book‐reading, play), the categories of MSL investigated (e.g., MSL, emotion terms) and the theoretical approach used. Such diversity in a relatively small sample of studies makes meta‐analysis challenging. A scoping review was also deemed more appropriate than a systematic review for this body of research, given that we aim to outline key concepts and detail the sources and types of experimental evidence available on this topic as opposed to offering conclusive answers regarding how parents use MSL based on their child's gender.

### Study selection

**FIGURE 1:**
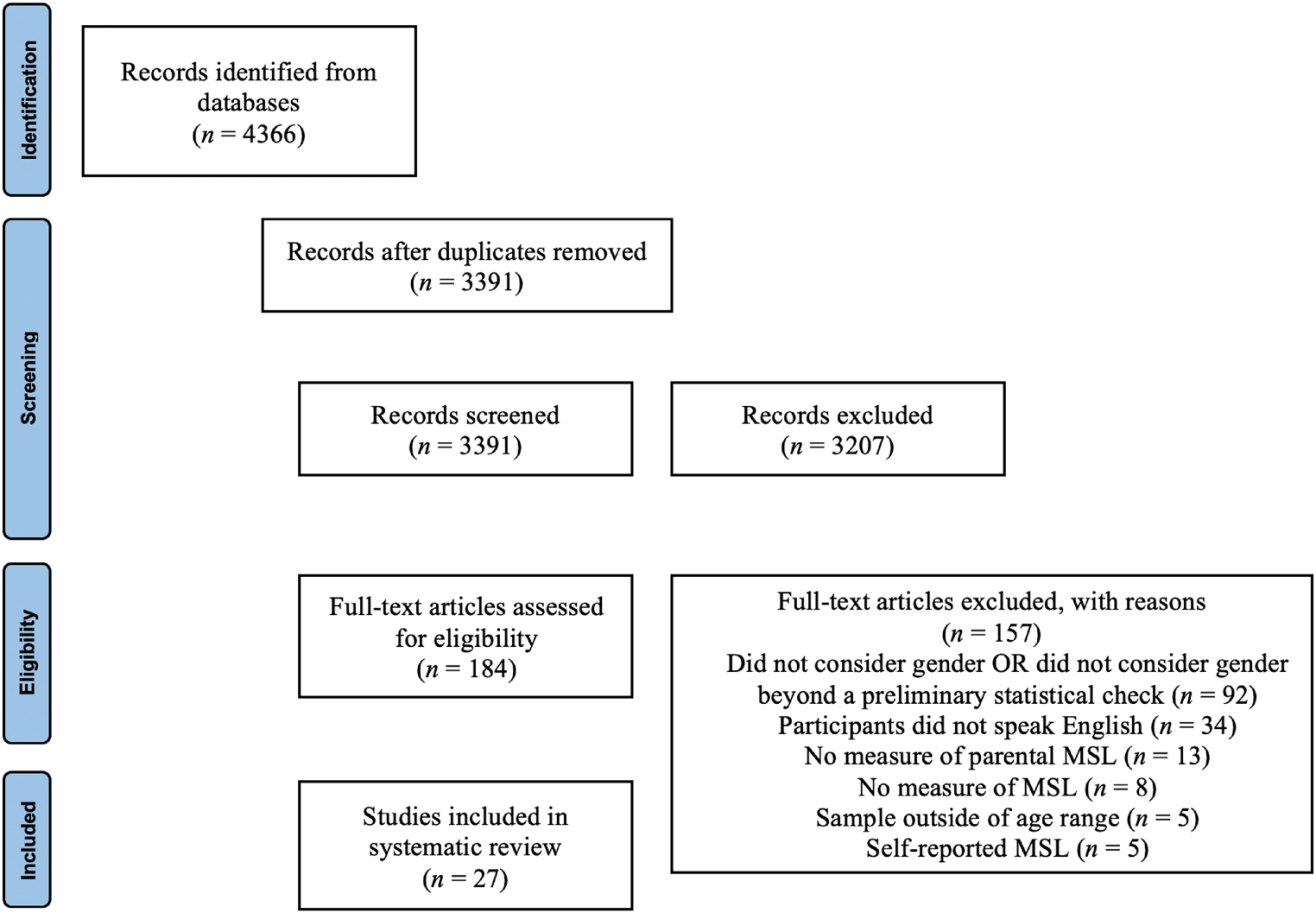
PRISMA flow diagram of the scoping review process.

All titles and abstracts were independently screened by CF (Reviewer 1 [R1]) and ES (Reviewer 2 [R2]) using Covidence review software following detailed training regarding the aims, inclusion and exclusion criteria of the review *reviewer initials blinded for review* (for details regarding Covidence software see https://app.covidence.org). Duplicate reports were excluded. Full texts were reviewed by R1 and R2 independently, and all extractions of those papers included were conducted independently by R1 and R2. Low tolerance of collaborative review was held to maintain consistency regarding all review decisions. In the instance reviewers R1 and R2 did not reach a consensus, the senior author assisted in reaching a final decision. Of the 4366 search result records screened, 184 studies were subsequently full‐text reviewed and 27 met the criteria for inclusion in the current review (see Figure 1 for details regarding the study selection process, including reasons for exclusion).

### Quality assessment

The quality of included studies was assessed using the Standard Quality Assessment Criteria (for the full criteria by which studies were scored, see Kmet et al., 2004). In short, the checklist for assessing the quality of quantitative studies, the inter‐rater agreement for overall scores of quantitative studies and the inter‐rater agreement for paper inclusion/exclusion using a variety of cut‐points for the overall scores in quantitative studies, as outlined by Kmet et al. (2004), were followed. All studies were independently scored by R1 and R2. Both sets of quality assessments (QA) can be found in Table 1. Agreement between R1 and R2 was acceptable (R1: *M* = 0.86, Range = 0.70–1.0; R2: *M* = 0.86, Range = 0.70–1.0; *r*(25) = 0.95, *p* < .001). Overall, the papers included in this review were acceptable or good quality.

**TABLE 1:** Characteristics of included studies.

Study	*N*	Participants reported as female (%)	Mean age (years)	Participants reported as white (%)	Country of study	QA R1	QA R2
1. Adams et al. (1995) L	24	47.06	3.33^1^, 5.83^2^	100.00	America	0.85	0.85
2. Brophy‐Herb et al. (2012)	123	52.85	2.37	85.00	America	0.90	0.90
3. Chang et al. (2017)	52	52.80	1.00	78.80	America	0.95	0.95
4. Doan and Wang (2010)	71	47.89	2.92	100.00	America	0.85	0.85
5. Drummond et al. (2014)	38	39.47	1.98	92.00	America	0.80	0.80
6. Dunn et al. (1987) L	43	51.16	1.50^1^, 2.00^2^	50.00	UK	0.80	0.80
7. Eisenberg (1999)	80	50.00	4.60	50.00	America	0.95	0.95
8. Howe and Rinaldi (2004)	32	50.00	3.87	100.00	Canada	0.90	0.90
9. Jenkins et al. (2003) L	40	50.00	4.40^1a^, 2.40^1b^; 6.30^2a^, 4.40^2b^	100.00	Canada	0.80	0.80
10. Jessee et al. (2016)	1114	49.28	2.00	77.80	America	1.0	0.95
11. Kuebli and Fivush (1992)	24	50.00	3.33	100.00	America	0.80	0.80
12. LaBounty et al. (2008)	106	50.94	3.42	93.00	America	0.90	0.95
13. Laible and Thompson (2002)	63	52.38	2.5^1^, 3^2^	79.00	America	0.85	0.85
14. Laible (2004)	51	54.90	4.08	77.00	America	0.80	0.80
15. Laible (2011)	50	54.00	4.22	84.00	America	0.85	0.85
16. Laranjo et al. (2014) L	59	57.63	1.06	78.90	Canada	0.90	0.95
17. Martin and Green (2005)	50	50.00	3.41	96.00	America	0.80	0.85
18. McElwain et al. (2011) L	1249	48	1.25^1^, 2^2^, 4.5^3^	77	America	0.95	0.90
19. Mcquaid et al. (2008)	33	42.42	4.75	100	Canada	0.85	0.85
20. Olson and Masur (2019) L	29	55.17	1.09^1^, 1.44^2^, 1.78^3^	93.10	America	0.75	0.75
21. Ontai and Thompson (2008)	78	53.85	4.48	NR	America	0.70	0.70
22. Pearson and Pillow (2016)	37	45.95	5.96^a^, 9.31^b^	78.90	America	0.85	0.85
23. Reynolds et al. (2020)	1164	50	0.17^1^, 0.5^2^	81	America	0.90	0.90
24. Roby and Scott (2018)	76	50.00	2.44	67.11	America	0.75	0.75
25. Roger et al. (2012)	57	50.88	2.71	85.70	Canada	1.0	1.0
26. Senehi et al. (2018)	95	51.58	2.16	88.40	America	0.90	0.90
27. Welch‐Ross et al. (1999)	32	53.13	4.14	NR	America	0.85	0.90

Abbreviations: L, Longitudinal Study; NR, Not Reported; QA, Quality Assessment.

^a,b^Experimental Group; ^1,2,3^Experimental Time Point.

### Data extraction and analysis

The following information was extracted for each included study: the number of child participants, percentage of child participants reported as girls within the sample, how participant gender was measured and operationalized, the mean age of child participants, the percentage of participants reported to be White within the sample, the country the data was collected in, the date of publication, the aim of the study, the results of the study that relate to the purpose of this review and if those results were significant or not. More complex information extracted from the included studies is explained in detail below.

#### Parental experimental involvement

The dyads of each study's sample were coded to represent mother–child dyads only, father–child dyads only, both mother–child and father–child dyads (meaning the child presented with both their mother and father) or a mixture of mother–child and father–child dyads (meaning that some dyads represent the former, some the later, but the child never presented with both their mother and father).

#### Experimental context

Following previous reviews (see Tompkins et al., 2018), we coded the experimental context used to elicit MSL, that is, the stimuli used to elicit MSL or the environment it was measured within. Context was coded as either reminiscing, picture/book stimuli, play, story‐based stimuli (e.g., a written vignette), observational or multiple combined contexts. Where a study used two or more of the contexts separately, more than one category was applied.

#### Measurement of MSL

Measurement refers to how MSL was measured experimentally, which means how specific (e.g., emotion terms) or broad (e.g., MSL generally) the study interest in MSL was. MSL measures were coded as one of two categories: (1) MSL (includes all forms of mental state talk, internal state talk, feeling terms and internal state language; e.g., emotion, cognitive and/or desire terms) and (2) emotion language (EL; includes emotion terms, emotion MSL, emotional discourse and references to emotion; e.g., emotion terms only).

Overall, the majority of studies (19 studies) used a broad MSL operationalization centred around parents' use of linguistic terms about cognitions, emotions and desires (see Table 2). While cognition, emotion and desire terms constituted most of these operationalizations, some studies included additional language categories. For example, Olson and Masur (2019) included parents' use of perception terms. In the case of Brophy‐Herb et al. (2012), modulations of assertion (e.g., perhaps, maybe) were included in a broad measurement of MSL.

**TABLE 2:** Methodological characteristics of the included studies.

Study	Measurement of MSL	Experimental context	Parental experimental involvement
1. Adams et al. (1995) L	EL	Reminiscing	Both Mothers and Fathers
2. Brophy‐Herb et al. (2012)	MSL	Picture/Book Stimuli	Mothers
3. Chang et al. (2017)	MSL	Story‐Based Stimuli	Mothers
4. Doan and Wang (2010)	MSL	Picture/Book Stimuli	Mothers
5. Drummond et al. (2014)	EL	Multiple Combined Contexts	Mixed Mothers and Fathers
6. Dunn et al. (1987) L	MSL	Observational	Mothers
7. Eisenberg (1999)	EL	Reminiscing	Mothers
8. Howe and Rinaldi (2004)	MSL	Observational and Picture/Book Stimuli	Mothers
9. Jenkins et al. (2003) L	MSL	Observational	Mixed Mothers and Fathers
10. Jessee et al. (2016)	MSL	Play	Mothers
11. Kuebli and Fivush (1992)	EL	Reminiscing	Both Mothers and Fathers
12. LaBounty et al. (2008)	MSL	Picture/Book Stimuli	Both Mothers and Fathers
13. Laible and Thompson (2002)	EL	Multiple Combined Contexts	Mothers
14. Laible (2004)	EL	Reminiscing and Picture/Book Stimuli	Mothers
15. Laible (2011)	EL	Reminiscing	Mothers
16. Laranjo et al. (2014) L	MSL	Play	Mothers
17. Martin and Green (2005)	EL	Story‐Based Stimuli	Mothers
18. McElwain et al. (2011) L	MSL	Picture/Book Stimuli and Play	Mothers
19. Mcquaid et al. (2008)	MSL	Story‐Based Stimuli	Mothers
Study	Measurement of MSL	Experimental Context	Parental Experimental Involvement
20. Olson and Masur (2019) L	MSL	Play	Mothers
21. Ontai and Thompson (2008)	MSL	Reminiscing	Mothers
22. Pearson and Pillow (2016)	MSL	Story‐Based Stimuli	Mothers
23. Reynolds et al. (2020) L	MSL	Picture/Book Stimuli and Play	Mixed Mothers and Fathers
24. Roby and Scott (2018)	MSL	Picture/Book Stimuli	Mixed Mothers and Fathers
25. Roger et al. (2012)	MSL	Picture/Book Stimuli	Both Mothers and Fathers
26. Senehi et al. (2018)	MSL	Picture/Book Stimuli	Mothers
27. Welch‐Ross et al. (1999)	MSL	Reminiscing	Mothers

Abbreviations: EL, Emotion Language; L, Longitudinal Study; MSL, Mental State Language.

## RESULTS

### Study characteristics

Characteristics of the 27 included studies are presented in Table 1. Importantly, studies primarily represented White American participants. The mean age of child participants in this review ranged from 0.17 to 9.31 years, and of the 4861 child participants, 50.50% were girls. Study sample sizes ranged from 24 to 1249 participants.

### Findings relating to parental MSL use and child gender

The majority of studies used a broad MSL operationalization. In contrast, eight used a narrower operationalization of a single category of MSL being emotion language (i.e., terms relating only to emotions; see Tables 2 and 3). Of the 27 included studies, 15 (55.56%) reported no significant association between child gender and parental MSL use. Of the 12 studies that *did* report a statistically significant association between ‘child gender and parents' MSL use, ten (83.33%) reported that girls received more parental MSL input towards them compared to boys, while two studies reported that boys received more parental MSL input compared to girls (see Table 3). Interestingly, of the ten studies that reported girls to receive more parental MSL compared to boys, four explicitly focused on parents' emotion language use. In contrast, the two studies that reported boys receive more parental MSL compared to girls focused on parents' MSL use more broadly.

**TABLE 3:** Summary of the relevant results and aims of included studies.

Study	Relevant study aim/s	Relevant findings	Result
1. Adams et al. (1995) L	To investigate gender differences in parental EL use	Mothers used more EL of increased diversity with girls than boys	M < F^a^
2. Brophy‐Herb et al. (2012)	Due to previous findings in the field, considered child gender	No differences in MSL use based on child gender	M = F
3. Chang et al. (2017)	To investigate whether the frequencies of maternal MSL differ by child gender	Mothers elaborated upon MSL terms more with girls than boys	M < F^a^
4. Doan and Wang (2010)	To investigate cultural differences in MSL use, including child gender	No differences in MSL use based on child gender	M = F
5. Drummond et al. (2014)	Due to previous findings in the field, considered child gender	Parents used a higher proportion of EL with girls than boys	M < F^a^
6. Dunn et al. (1987) L	To investigate gender differences in feeling term use	At 18 months and 24 months mothers used more feeling terms with girls than boys	M < F^a^
7. Eisenberg (1999)	To investigate if mother‐daughter dyads use more EL than mother‐son dyads	Mothers used more EL of increased diversity with girls than boys	M < F^a^
8. Howe and Rinaldi (2004)	To explore associations between gender and maternal MSL use	Mothers used more MSL during leave‐taking with girls than boys	M < F^a^
9. Jenkins et al. (2003) L	Due to previous findings in the field, considered child gender	No differences in MSL use based on child gender	M = F
10. Jessee et al. (2016)	Due to previous findings in the field, considered child gender	Mothers used more desire and emotion terms with girls than boys	M < F^a^
11. Kuebli and Fivush (1992)	Due to previous findings, mothers were predicted to use more EL with girls	Parents used more EL of increased diversity with girls than boys	M < F^a^
12. LaBounty et al. (2008)	Aimed to investigate mothers' and fathers' MSL use towards their children	Mothers used more cognitive and desire terms with girls than boys	M < F^a^
13. Laible and Thompson (2002)	Due to previous findings in the field considered child gender	No differences in EL use based on child gender	M = F
14. Laible (2004)	Due to previous findings in the field, considered child gender	No differences in EL use based on child gender	M = F
15. Laible (2011)	To investigate child‐level differences in EL use	No differences in EL use based on child gender	M = F
16. Laranjo et al. (2014) L	Gender differences were investigated in an exploratory way regarding MSL use	No differences in MSL use based on child gender	M = F
17. Martin and Green (2005)	Predicted that mothers' EL use may not differ with girls and boys	No differences in MSL use based on child gender	M = F
18. McElwain et al. (2011) L	Due to previous findings in the field, considered child gender	No differences in MSL use based on child gender	M = F
19. Mcquaid et al. (2008)	Gender differences were investigated in an exploratory way regarding MSL use	Mothers used more MSL with girls than boys	M < F^a^
20. Olson and Masur (2019) L	Due to previous findings in the field, considered child gender	No differences in MSL use based on child gender	M = F
21. Ontai and Thompson (2008)	Due to previous findings in the field, considered child gender	No differences in MSL use based on child gender	M = F
22. Pearson and Pillow (2016)	Due to previous findings in the field, considered child gender	No differences in MSL use based on child gender	M = F
23. Reynolds et al. (2020) L	Due to previous findings in the field, considered child gender	No differences in MSL use based on child gender	M = F
24. Roby and Scott (2018)	No specific hypotheses but considered gender as a variable.	No differences in MSL use based on child gender.	M = F
25. Roger et al. (2012)	Gender differences were investigated in an exploratory way regarding MSL use.	Parents used more MSL with boys than girls.	M > F^a^
26. Senehi et al. (2018)	No specific hypotheses but considered gender as a variable	No differences in MSL use based on child gender	M = F
27. Welch‐Ross et al. (1999)	Due to previous findings in the field, considered child gender	Mothers used more MSL with boys than girls	M > F^a^

Abbreviations: EL, Emotion Language; L, Longitudinal Study; MSL, Mental State Language.

^a^ Significant finding relevant to current review.

### Measurement and operationalization of gender

The measurement of gender within the included studies can be considered homogenous. None of the studies provided a formal definition or operationalization of gender, so presumably, researchers relied on parental reporting of a child's assigned sex at birth. Further, only binary categories of child gender were reported (girls and boys). Importantly, due to the lack of a formal definition or operationalization of gender, it is assumed that researchers employed commonly used demographics questions (e.g., what is your child's sex/gender?) to collect such information. Additionally, no studies reported measuring a child's or parents' gender identity or expression.

### Aims relating to parental MSL use and child gender

Across the reviewed literature, a number of broad aims motivated researchers to consider the association between child gender and parental MSL use. Overall, four studies actively investigated child gender as a variable to account for any impact it may have had on experimental variables (including MSL) and developmental trajectories, meaning the examination was not necessarily motivated by any theoretical preconception nor the findings of previous work (see Table 3).

The majority of studies aimed to consider gender simply due to previous findings related to the association between gender and MSL, regardless of significance (see Table 3). This is a common motivation for experimental work in child development and is likely motivated by the mixed and inconsistent findings concerning the association between child gender and parental MSL. For example, Pearson and Pillow (2016) cite previous findings regarding gender differences in mother–child conversations about emotions as the motivation for considering if mothers used MSL differently based on a child's gender.

Several studies explicitly stated that the study aimed to investigate differences in MSL use as a result of child gender due to theoretically or experimentally grounded reasoning (see Table 3). Interestingly, Adams et al. (1995) considered how previous findings regarding associations between emotion socialization and gender may be reflected in parents' use of emotion language and the specificity of emotion terms. LaBounty et al. (2008) aimed to investigate differences in mothers' and fathers' MSL use towards their children. However, as a part of this, the gender of the child was also considered due to previous research findings regarding this association.

Lastly, four studies investigated associations between child gender and parental MSL use in an exploratory way that may have explained another outcome variable of interest (see Table 3). For example, Laranjo et al. (2014) examined if an association between child gender and parental MSL use may have explained gender differences in attachment security.

### Parent experimental involvement

Maternal MSL use has been the most frequently researched MSL input to children in the last three decades (see Tompkins et al., 2018). This is likely because even within contemporary society, women typically provide the most housework, parenting and care within a family (Mullens & Glorieux, 2023). This trend is reflected within the current review, with 19 of 27 studies only considering the MSL use of mothers. Four studies measured the MSL use of both mothers and fathers. All four of these studies reported a significant association between child gender and parental MSL use. Lastly, four further studies measured the MSL use of a mixture of mothers and fathers, but never both the mother and father of a single child participant (see Table 2).

### Experimental context

The context used to illicit parents' MSL is associated with the amount of MSL produced (see Tompkins et al., 2018, for a review). Further, many of these contexts can contain highly gendered and salient objects, protagonists and social activities. For example, a play context may contain multiple toys that can be considered stereotypically feminine (i.e., dolls, baking sets) or a book context may employ a book that depicts a feminine protagonist doing stereotypically feminine activities (i.e., playing with dolls, baking cakes). Therefore, it is important to consider this methodological factor when reviewing the literature regarding child gender and parental MSL use.

A variety of contexts were employed by the studies included in this review. Six of the 27 studies used a picture or book stimuli to elicit MSL, six studies used reminiscing to elicit MSL, four studies used a written or verbal story, three studies used to play to elicit MSL, and two studies measured parental MSL use within a naturalistic observational context. Six studies used multiple combined contexts to elicit MSL (see Table 2). For studies that reported significant findings on the relationship between parental MSL and a child's gender, reminiscing was most frequently used, with four of 12 studies employing this context. In contrast, those studies that reported non‐significant findings most frequently used picture or book stimuli or multiple combined contexts (see Tables 2 and 3).

## DISCUSSION

This scoping review examines the findings of previous works investigating associations between child gender and parents' MSL use. Twenty‐seven studies met the inclusion criteria for this review. Several decades of research have focused on the importance and role of MSL in the development. However, it is surprising that so few studies have explicitly considered the relationship that child gender may have with parental MSL. When gender *is* considered, it may be done so in a way that relies on the assumed assigned sex at birth. Additionally, MSL measurement is approached in a variety of ways, making it difficult to draw firm conclusions regarding the association between child gender and parental MSL use.

### Child gender and parental MSL use

Findings regarding the association between child gender and parental MSL use were mixed. Twelve of 27 studies reported a significant association, with 10 indicating that girls receive more parental MSL input than boys and two studies reporting the opposite significant pattern (see Table 3). Although this suggests a greater propensity to use MSL when speaking with girls compared to boys, the majority of included studies here report no associations between child gender and parents' MSL. Therefore, the evidence is far from conclusive.

Although the evidence is inconclusive, this review has value in systematically compiling and reviewing the evidence to date regarding the association between child gender and parental MSL use. This is a valuable synthesis, which highlights the need for future researchers to consider how they operationalize and measure both gender and MSL. Additionally, this review may encourage researchers to reflect on methodological choices prior to designing new studies on this topic, specifically in regard to the context in which they measure MSL, the gendered stimuli that may be present in these contexts and the approach they take to parental involvement (the study of just mothers, mothers and fathers or both). It is possible that the findings remain inconsistent to date due to the fact that researchers have failed to consider these factors when investigating the associations between child gender and parents' MSL.

The continued assessment of potential gender differences in parental MSL use is important, given that MSL plays a crucial role in the development of a child's ToM (Ruffman, 2023; Slaughter, 2015; Tompkins et al., 2018), emotion understanding (Garner et al., 1997), emotion vocabulary (Adams et al., 1995) and general MSL vocabulary (Ruffman et al., 2023). From a sociocultural and ecological theoretical perspective (Bronfenbrenner & Morris, 1998; Leaper, 2002; Leaper & Friedman, 2007), if girls *do* receive more MSL (in line with the general pattern of significant findings observed in this scoping review), this may reflect societal stereotype beliefs and gender roles. Parents may view children who express gender in a feminine way as more likely to attend to, and be interested in, the mental states of social agents. Further, parents may view girls as accruing more beneficial outcomes throughout development by being exposed to talk about mental states (especially those relating to emotions) compared to boys. For example, exposure to such language may aid girls in fulfilling societal roles that are stereotypically expected of them now or in the future (Eagly & Wood, 2012). Thus, parents may unconsciously reinforce gender stereotypes through their use of MSL.

Gender Schema Theory posits that gender development is closely tied to cognitive processes. Children develop gender schemas to aid their cognitive understanding of being a girl or boy, influencing their behaviours and information processing. These schemas develop and change over time, influenced by interactions within their social contexts. Children actively shape their gender development by processing information. Here, schemas are active constructs children use to seek or understand information (Martin et al., 2002; Martin & Halverson, 1981). In the context of the findings of this review, Gender Schema Theory would suggest that parents may subconsciously reinforce gender stereotypes through emotion language and MSL (i.e., because girls are more emotionally attuned), reinforcing a child's schemas about their own and other's gender. However, this association must be viewed in light of limitations, which include variations in MSL measurement, the measurement of gender and factors related to the experimental design of studies included in this review. Hence, questions remain regarding the association between child gender and parents' MSL use.

### Measurement and operationalization of gender

It is assumed that all included studies relied on assigned sex at birth and did not include measures of child or parent gender, as this was not reported. Using assigned sex at birth as a proxy for a measure of child gender may further conflate sex and gender as interchangeable terms. Broadly, science has for some time acknowledged that sex and gender are different but sometimes related constructs, with the former representing biological systems and the latter being socially constructed (see Deaux, 1985; Fausto‐Sterling et al., 2012; Torgrimson & Minson, 2005). The use of a proxy or undefined gender measure fails to consider the nuances and differences that may exist between a child's gender expression, gender identity and biological sex, or how a child's gender expression and identity may impact parents' use of MSL when interacting with that child, over and above their biological sex (Martin et al., 2002; Ruble et al., 2007; Ruble & Martin, 1998). The studies included here do not clearly define what is meant by terms such as ‘gender’, ‘sex’, ‘girl’ or ‘boy’, for example. Such unclear operationalizations risk misinterpretation by consumers of research, or for findings to perpetuate stereotypes regarding gender or sex differences in parents' use of MSL, possibly influencing the MSL that parents use towards their children and creating less developmentally rich linguistic environments for children of certain genders. There is no reason that future research cannot measure both parent and child sex and gender where appropriate, using clear operationalizations and informed measures.

Age‐appropriate measures of child gender identity have been developed recently. For example, Gülgöz et al.s' (2022) continuous measure of gender identity can be used with children from a young age and allows for responses outside of the gender binary. The employment of informed and clearly defined gender measurement may allow the field to better test the associations between parental MSL use and child gender. If both sex and gender are considered, it may be possible to disentangle the biological (sex) versus social (gender) influences of such associations, furthering our understanding of the influence that child gender has on parental MSL use.

Additionally, updating the measurement of gender in this context allows for the inclusion of gender‐diverse children. Demonstrating this approach, Farrell et al. (2023) examined adults' self‐reported preferences for MSL and elaborated language towards feminine, masculine and gender‐neutral child protagonists, finding that adults preferred to use MSL less with masculine child protagonists and elaborated language less with gender‐neutral child protagonists (Farrell et al., 2023). Currently, no research to date explores parents' MSL use towards children who are gender diverse in an observational context. This limits how generalizable the findings presented in this scoping review are to gender‐diverse children and their parents. Future research should aim to overcome such limitations due to the influence of MSL on children's social‐cognitive development.

### Aims relating to parental MSL use and child gender

As presented in this review, motivations to consider the association between child gender and parental MSL varied. Most included studies aimed to consider this association due to the previous findings of prior published studies. We suggest that researchers should clearly articulate the theoretical and empirical rationale behind including child gender as a variable in future research. Additionally, we urge researchers to avoid simply controlling for child gender in future work because it may present a potential confound. Rather, they should consider gender a key variable that can offer insights into how children develop within linguistic environments that may differ based on their gender (see Shapiro et al., 2021, for a review on this topic).

### Parent experimental involvement

Maternal MSL use has been the primary research focus for several decades. Hence, it is unsurprising that 19 of the 27 included studies in this review considered the MSL used by mothers. In contrast, four studies examined the MSL use of a mixture of mothers and fathers, but not both, and four studies examined the MSL use of both mothers and fathers (see Table 2). Generally, these studies found that mothers' and fathers' MSL use did not differ. However, LaBounty et al. (2008) reported that mothers used more MSL than fathers. Notably, all four studies that included mothers and fathers reported a significant association between child gender and parental MSL use. Based on the few studies that report inconsistent outcomes discussed here, it is clear that more work is required to understand if there are differences in mothers' and fathers' MSL use and if any existing differences are influenced by their own gender or that of their child.

The limited inclusion of fathers in the studies presented here may restrict the generalizability of the findings. Investigating how fathers use MSL is essential for a comprehensive understanding of how adults use MSL towards children based on the child's gender. This is particularly relevant to the study of gender development, given that it has been suggested that fathers are more likely than mothers to socialize children to fulfil traditional gender roles (Coltrane & Adams, 1997; Maccoby, 1998). Future research investigating parents' MSL use and its association with child gender should aim to recruit comparable samples of fathers and mothers where relevant. Exploring the contributions of different caregivers may provide insights into adult MSL use and how we socialize children's gender identities through language.

### Experimental context

Researchers have acknowledged contextual influences on the elicitation of parents' MSL (Tompkins et al., 2018; Ziv et al., 2013). Within the reviewed studies, the choice of context and how it may be gendered or contain gendered stimuli was rarely considered nor discussed. However, context has been demonstrated to influence parental use of MSL within some of the reviewed studies (e.g., Laible, 2004; Laranjo et al., 2014; Meins et al., 2013), further emphasizing its importance as a methodological consideration. The studies included in the present review used a variety of contexts to elicit parents' MSL. The distribution of findings across different contexts suggests the need for further investigation to understand if associations between child gender, MSL and specific elicitation contexts exist and, if so, which contexts give rise to certain associations between child gender and parental MSL. It is plausible to hypothesize that reminiscing contexts sometimes involve the recounting of an emotional experience that places the child or parent at the centre (e.g., Welch‐Ross et al., 1999), thus attracting the gendered expectations that girls and mothers have experienced better emotional socialization and are therefore more likely to comprehend mental state talk about emotions or emotional states (see Denham et al., 2010). However, such hypotheses require experimental investigations that employ carefully designed and considered contexts to elicit parents' MSL.

### Measurement and operationalization of MSL

This review demonstrates heterogeneity in the operationalization and subsequent measurement of MSL, ranging from broad definitions inclusive of several mental states to narrow definitions focused on a single mental state (e.g., emotion terms). Most studies herein employed a broad operationalization that captured cognition, emotion and desire terms within a single category (i.e., MSL). However, the majority of studies that reported parents used greater amounts of MSL with girls found this association specifically in relation to desire and emotion terms (see Table 3). Perhaps the reliance on a broad operationalization of MSL within the reviewed literature has less sensitivity to detect differences in parental MSL use as a function of child gender.

Research has underscored the developmental significance of specific features of MSL content and quality. For example, mothers' use of cognitive terms, in particular, has been associated with children's ToM ability (Adrian et al., 2005; Slaughter et al., 2007; Taumoepeau & Ruffman, 2008). Mothers' elaborated talk regarding mental states has been strongly associated with children's ToM ability, in contrast to simple mentions (Ontai & Thompson, 2008; Slaughter et al., 2007). Additionally, the appropriateness of parents' descriptions or explanations of mental states has been reported to influence a child's ToM ability (Meins et al., 2013; Tompkins et al., 2018).

Hence, future investigations should aim to understand if features of MSL (e.g., cognitive terms) and MSL quality (e.g., elaboration or appropriateness) shift as a function of child gender and if these features of MSL are impacted by the gendering of the context in which they are measured. For example, cognitive terms may be used more with boys in STEM‐based contexts, and emotion terms may be used more with girls in caregiving‐based contexts. Further exploration of these nuances would increase our understanding of what categories of MSL may be used in developmental contexts depending on the child's gender.

### Limitations and future directions

One strength of this review is the broad and systematic search, which likely captured all relevant primary studies published in peer‐reviewed academic journals. However, the search did not consider chapters, grey literature or unpublished material, which may have resulted in relevant studies (especially those reporting non‐significant results) not being included in the current review. Another limitation is that only studies that recorded participants' use of MSL in English were included. Subsequently, the findings presented may not generalize to populations that speak other languages. Most participants within the included studies in this review were White, limiting the generalizability of the findings offered here. Future research exploring the association between parents' MSL and child gender should strongly consider diversifying samples. Further, researchers should consider the possibility of cross‐cultural projects, such as the work by Chang et al. (2017), considering the MSL use of U.S. American and Chilean mothers.

Prior research has demonstrated that parents may vary their MSL use towards their children as a function of that child's age (see Ruffman et al., 2002; Taumoepeau & Ruffman, 2008). Additionally, children's gender identities develop throughout their lifespan, with gender identity first forming around the age of 3 years (for a detailed review see Perry et al., 2019). Therefore, it is possible that the association between child gender and parental MSL use may change over time. For example, parents may adapt their MSL to complement or even counteract their children's gender socialization. Further, parents may use MSL to discuss emotions with boys and girls differently only when the child begins to display and express gender in a salient way. Thus, although it was not the focus of this review, the relationship between child age, gender and parents’ MSL use should be explored in future studies and reviews. This may improve our understanding of whether and how the association between child gender and parental MSL use varies as a function of age.

The current scoping review aimed to synthesize studies that explicitly investigated the association between child gender and parental MSL use. Therefore, included studies were required to consider child gender as a participant variable in the study design, rather than a preliminary test. We acknowledge that this approach may have biased the review towards studies reporting significant gender differences. However, our aim was not to exhaustively collate findings, as per a systematic review or meta‐analysis. Rather our aim was to examine studies that investigated the association between child gender and parental MSL in a meaningful and considered way and to highlight gaps in the evidence base. Based on the current findings, we suggest that a systematic review and meta‐analysis of this topic, incorporating broader inclusion criteria, would be warranted.

Additionally, it is notable that previous research has found gender‐specific patterns in parents' use of emotion language. For example, mothers' discussions of sadness have been found to be more common and elaborated when speaking with girls. In contrast, discussion about anger is more common with boys (e.g., Fivush, 1991; Fivush et al., 2000). We recognize the significance of such findings and their potential impact on children's gender and emotion socialization. However, the current review aimed to investigate parents’ broader use of MSL and its association with child gender. Further, valence only applies to emotion terms, while we conceptualize MSL here as language consisting of cognitive, desire and emotion terms. Regardless, by not considering the valence of parent's emotion language, we were unable to review the nuanced relationships between children's gender and parent's emotion language valence, therefore limiting the conclusions we are able to draw here. By considering the valence of emotion language, future work may enrich our understanding of the complex associations between language, gender and socialization processes. Further, future investigations that incorporate a multidimensional approach to studying parental language practices can offer deeper insights into the mechanisms underlying gendered patterns of communication within developmental contexts.

## CONCLUSION

The findings regarding the association between parents' MSL and child gender reported here are mixed. While the majority of studies report no difference in parents' use of MSL as a function of child gender, those that do report an effect indicate greater MSL when parents speak to girls. However, the heterogeneous measurement of MSL and the limited measurement of gender do not allow for definitive conclusions from the literature presented here. Therefore, areas of improvement have been identified for future research. By applying these recommendations, especially regarding the measurement and operationalization of gender and MSL, along with methodological considerations and motivations for investigation, future work should provide more considered and informative evidence regarding the association between child gender and parents' MSL use. This review suggests that scientists and practitioners should exercise caution when making assumptions regarding gender differences based on limited or inconsistent bodies of evidence, such as that presented here. Generalizing or simplifying findings can perpetuate gender stereotypes and discount the experiences of children and parents alike. Collectively, as a field, we should strive to achieve a more accurate and nuanced understanding of the influence of children's gender on linguistic, developmental and social inputs. By doing so, we will hopefully promote safe, informed and developmentally rich spaces for all children, regardless of their gender.

## AUTHOR CONTRIBUTIONS

**Callyn Farrell:** Conceptualization; writing – original draft; writing – review and editing; project administration; investigation; methodology; data curation; formal analysis. **Ellen Sanderson:** Methodology; data curation. **Aisling Mulvihill:** Conceptualization; writing – review and editing; supervision. **Michael Thai:** Conceptualization; writing – review and editing; supervision. **Virginia Slaughter:** Conceptualization; writing – review and editing; supervision.

## FUNDING INFORMATION

The current project received no specific funding.

## CONFLICT OF INTEREST STATEMENT

The authors have no conflicts of interest to declare.

## Supporting information


Appendix S1

## Data Availability

No data were used in this review. However, searches can be replicated using the information provided in the supplementary materials.
