# Supplementary material for: Parents' mental state language and child gender: A scoping review of developmental evidence
Source: Br J Dev Psychol. 2024 Jun 6;43(2):396–417. doi: 10.1111/bjdp.12502 (PMC12070136; doi:10.1111/bjdp.12502)
Supplement: Supplementary file 1 — Appendix S1 [file BJDP-43-396-s001.docx]

**Appendix A**

Search Information

**Search terms**

| “mental state*” OR “mental-state*” OR “internal state*” OR “internal-state*” OR “mental verb” OR “emotion* term” OR “emotion* vocab*” OR “cognitive state*” OR “cognitive reference” OR “maternal state*” OR “maternal-state*” OR “desire term*” |
| --- |
| AND |
| language* OR vocab* OR convers* OR discuss* OR talk* OR discours* |
| AND |
| parent* OR mother* OR maternal* OR father* OR paternal* OR adult* OR child* OR caregiver* OR famil* OR infan* |

**Filters, limits, and date of search**

**PsycINFO**

- 24/01/2023
- Upper age filter of 12 years applied

**Scopus**

- 24/01/2023
- Search limited to title/abstract and psychology filter applied

**ERIC**

- 24/01/2023
- Limited to NOFT

**Google Scholar**

- 24/01/2023
- 19,500 results returned (all search words in “at least one of the words”, anywhere in the article selected, and limited to the year range of 1980-2021). The first 597 results were selected and exported for screening.

**Appendix B**

References for those studies included in the current scoping review

1. Adams, S., Kuebli, J., Boyle, P. A., & Fivush, R. (1995). Gender differences in parent-child conversations about past emotions: A longitudinal investigation. *Sex Roles, 33*(5-6), 309–323. <https://doi.org/10.1007/BF01954572>
2. Brophy-Herb, H. E., Stansbury, K., Bocknek, E., & Horodynski, M. A. (2012). Modeling maternal emotion-related socialisation behaviors in a low-income sample: Relations with toddlers’ self-regulation. *Early Childhood Research Quarterly*, *27*(3), 352–364. <https://doi.org/10.1016/j.ecresq.2011.11.005>
3. Chang, T.-F., Farkas, C., Vilca, D., & Vallotton, C. (2017). U.S. and Chilean mothers’ use of mental references with infant girls and boys: Comparison of maternal practices in gender socialisation via language in two countries. *Journal of Cross-Cultural Psychology*, *48*(8), 1271–1287. <https://doi.org/10.1177/0022022117720752>
4. Doan, S. N., & Wang, Q. (2010). Maternal discussions of mental states and behaviors: Relations to emotion situation knowledge in European American and immigrant Chinese children. *Child Development, 81*(5), 1490–1503. <https://doi.org/10.1111/j.1467-8624.2010.01487.x>
5. Drummond, J., Paul, E. F., Waugh, W. E., Hammond, S. I., & Brownell, C. A. (2014). Here, there and everywhere: Emotion and mental state talk in different social contexts predicts empathic helping in toddlers. *Frontiers in Psychology*, *5*, 361–361. <https://doi.org/10.3389/fpsyg.2014.00361>
6. Dunn, J., Bretherton, I., & Munn, P. (1987). Conversations about feeling states between mothers and their young children. *Developmental Psychology*, *23*(1), 132–139. <https://doi.org/10.1037/0012-1649.23.1.132>
7. Eisenberg, A. R. (1999). Emotion talk among Mexican American and Anglo American mothers and children from two social classes. *Merrill-Palmer Quarterly, 45(*2), 267–284.
8. Howe, N., & Rinaldi, C. M. (2004). 'You be the big sister': Maternal-preschooler internal state discourse, perspective-taking, and sibling caretaking. *Infant and Child Development*, *13*(4), 217–234. <https://doi.org/10.1002/icd.350>
9. Jenkins, J. M., Turrell, S. L., Kogushi, Y., Lollis, S., & Ross, H. S. (2003). A longitudinal investigation of the dynamics of mental state talk in families. *Child Development*, *74*(3), 905–920. <https://doi.org/10.1111/1467-8624.00575>
10. Jessee, A., McElwain, N. L., & Booth-LaForce, C. (2016). Maternal supportive behavior, cognitive talk, and desire/emotion talk at 24 months: Distinct factors and differential antecedents. *Parenting, Science and Practice, 16*(2), 63–86. <https://doi.org/10.1080/15295192.2016.1134988>
11. Kuebli, J., & Fivush, R. (1992). Gender differences in parent-child conversations about past emotions. *Sex Roles, 27*(11-12), 683–698. <https://doi.org/10.1007/BF02651097>
12. LaBounty, J., Wellman, H. M., Olson, S., Lagattuta, K., & Liu, D. (2008). Mothers' and Fathers' use of internal state talk with their young children. *Social Development, 17*(4), 757–775. <https://doi.org/10.1111/j.1467-9507.2007.00450.x>
13. Laible, D., & Thompson, R. A. (2002). Mother-child conflict in the toddler years: Lessons in emotion, morality, and relationships. *Child Development, 73*(4), 1187–1203. <https://doi.org/10.1111/1467-8624.00466>
14. Laible, D. (2004). Mother-child discourse in two contexts: Links with child temperament, attachment security, and socioemotional competence. *Developmental Psychology, 40*(6), 979–992. <https://doi.org/10.1037/0012-1649.40.6.979>
15. Laible, D. (2011). Does it matter if preschool children and mothers discuss positive vs. negative events during reminiscing? Links with mother-reported attachment, family emotional climate, and socioemotional development. *Social Development, 20*(2), 394–411. <https://doi.org/10.1111/j.1467-9507.2010.00584.x>
16. Laranjo, J., Bernier, A., Meins, E., & Carlson, S. M. (2014). The roles of maternal mind-mindedness and infant security of attachment in predicting preschoolers’ understanding of visual perspective taking and false belief. *Journal of Experimental Child Psychology, 125*(1), 48–62. <https://doi.org/10.1016/j.jecp.2014.02.005>
17. Martin, R. M., & Green, J. A. (2005). The use of emotion explanations by mothers: Relation to preschoolers' gender and understanding of emotions. *Social Development, 14*(2), 229–249. <https://doi.org/10.1111/j.1467-9507.2005.00300.x>
18. McElwain, N. L., Booth-LaForce, C., & Wu, X. (2011). Infant-mother attachment and children's friendship quality: Maternal mental-state talk as an intervening mechanism. *Developmental Psychology, 47*(5), 1295–1311. <https://doi.org/10.1037/a0024094>
19. Mcquaid, N., Bigelow, A. E., McLaughlin, J., & MacLean, K. (2008). Maternal mental state language and preschool children's attachment security: Relation to children's mental state language and expressions of emotional understanding. *Social Development,* 17(1), 61–83. <https://doi.org/10.1111/j.1467-9507.2007.00415.x>
20. Olson, J., & Masur, E. F. (2019). Developmental changes in the frequency and complexity of mothers’ internal state utterances across the second year. *First Language, 39*(4), 462–476. <https://doi.org/10.1177/0142723719850001>
21. Ontai, L. L., & Thompson, R. A. (2008). Attachment, parent-child discourse and theory-of-mind development. *Social Development, 17*(1), 47–60. <https://doi.org/10.1111/j.1467-9507.2007.00414.x>
22. Pearson, R. M., & Pillow, B. H. (2016). Mother-child conversation and children's social understanding during middle childhood. *The Journal of Genetic Psychology, 177*(4), 103–121. <https://doi.org/10.1080/00221325.2016.1188054>
23. Reynolds, E., Garrett‐Peters, P., Bratsch‐Hines, M., & Vernon‐Feagans, L. (2020). Mothers' and fathers' mental state talk: Ethnicity, partner talk, and sensitivity. *Journal of Marriage and Family, 82(*5), 1696–1716. <https://doi.org/10.1111/jomf.12675>
24. Roby, E., & Scott, R. M. (2018). The relationship between parental mental-state language and 2.5-year-olds’ performance on a nontraditional false-belief task. *Cognition, 180*, 10–23. <https://doi.org/10.1016/j.cognition.2018.06.017>
25. Roger, K. M., Rinaldi, C. M., & Howe, N. (2012). Mothers' and fathers' internal state language with their young children: An examination of gender differences during an emotions task. *Infant and Child Development, 21*(6), 646–666. <https://doi.org/10.1002/icd.1762>
26. Senehi, N., Brophy-Herb, H. E., & Vallotton, C. D. (2018). Effects of maternal mentalisation-related parenting on toddlers' self-regulation. *Early Childhood Research Quarterly, 44*, 1–14. <https://doi.org/10.1016/j.ecresq.2018.02.001>
27. Welch-Ross, M. K., Fasig, L. G., & Farrar, M. J. (1999). Predictors of preschoolers' self-knowledge: Reference to emotion and mental states in mother-child conversation about past events. *Cognitive Development, 14*(3), 401–422. <https://doi.org/10.1016/S0885-2014(99)00012-X>
